# Regulator-dependent temporal dynamics of a restriction-modification system's gene expression upon entering new host cells: single-cell and population studies

**DOI:** 10.1093/nar/gkab183

**Published:** 2021-03-21

**Authors:** Alessandro Negri, Olesia Werbowy, Ewa Wons, Simon Dersch, Rebecca Hinrichs, Peter L Graumann, Iwona Mruk

**Affiliations:** Department of Microbiology, Faculty of Biology, University of Gdansk, Wita Stwosza 59, Gdansk 80–308, Poland; Department of Microbiology, Faculty of Biology, University of Gdansk, Wita Stwosza 59, Gdansk 80–308, Poland; Department of Microbiology, Faculty of Biology, University of Gdansk, Wita Stwosza 59, Gdansk 80–308, Poland; SYNMIKRO, LOEWE Center for Synthetic Microbiology, Marburg, Germany; Department of Chemistry, Philipps Universität Marburg, Hans-Meerwein-Strasse 6, 35032 Marburg, Germany; SYNMIKRO, LOEWE Center for Synthetic Microbiology, Marburg, Germany; Department of Chemistry, Philipps Universität Marburg, Hans-Meerwein-Strasse 6, 35032 Marburg, Germany; SYNMIKRO, LOEWE Center for Synthetic Microbiology, Marburg, Germany; Department of Chemistry, Philipps Universität Marburg, Hans-Meerwein-Strasse 6, 35032 Marburg, Germany; Department of Microbiology, Faculty of Biology, University of Gdansk, Wita Stwosza 59, Gdansk 80–308, Poland

## Abstract

Restriction-modification (R-M) systems represent a first line of defense against invasive DNAs, such as bacteriophage DNAs, and are widespread among bacteria and archaea. By acquiring a Type II R-M system via horizontal gene transfer, the new hosts generally become more resistant to phage infection, through the action of a restriction endonuclease (REase), which cleaves DNA at or near specific sequences. A modification methyltransferase (MTase) serves to protect the host genome against its cognate REase activity. The production of R-M system components upon entering a new host cell must be finely tuned to confer protective methylation before the REase acts, to avoid host genome damage. Some type II R-M systems rely on a third component, the controller (C) protein, which is a transcription factor that regulates the production of REase and/or MTase. Previous studies have suggested C protein effects on the dynamics of expression of an R-M system during its establishment in a new host cell. Here, we directly examine these effects. By fluorescently labelling REase and MTase, we demonstrate that lack of a C protein reduces the delay of REase production, to the point of being simultaneous with, or even preceding, production of the MTase. Single molecule tracking suggests that a REase and a MTase employ different strategies for their target search within host cells, with the MTase spending much more time diffusing in proximity to the nucleoid than does the REase. This difference may partially ameliorate the toxic effects of premature REase expression.

## INTRODUCTION

Bacteria have evolved a repertoire of defense mechanisms against mobile genetic elements, such as bacteriophages, plasmids and transposons (1). The most prevalent and efficient ones seem to be restriction-modification (R-M) systems and CRISPRs (2), though many other such systems appear to exist as well (3). These modules can limit the flux of genetic material into host cells, strongly impacting bacterial genomes (4–8). In addition, R-M systems may also modulate this process by facilitating the acquisition of foreign DNA (9,10). R-M systems and CRISPRs are mobile themselves (9,11–14).

Among the four main Types of R-M systems, Type II is the most described so far and the simplest in structure. Most of them include two independent enzymes, a restriction endonuclease (REase) and a DNA methyltransferase (MTase). Both enzymes recognize the same short DNA sequence, which the MTase modifies by adding a methyl group, while the cognate REase cleaves it unless methylated (15). While not discussed further here, there are some REases that only cleave a target sequence if it is methylated (16).

The acquisition of an R-M system by a new host cell could lead to at least two new features, that are not mutually exclusive: (i) MTase action can impact the epigenetic status, affecting gene expression and leading to new phenotypes (17–27); (ii) REase action can provide a potent anti-invading DNA mechanism; (iii) their regulation can lead to host genome damage or post-segregational cell killing, due to the toxic nature of REases in the absence of sufficient protective methylation (28,29).

Balancing REase and MTase gene expression is crucial to regulate genome destruction by the potentially-toxic REase, and several regulatory strategies have been reported (28). These strategies include: regulatory MTases that bind operator sequences (30–32), antisense RNAs (33,34), and dedicated transcription factors called C proteins (35–40) (first reported for the PvuII R-M system; (41)). Some C proteins have been structurally characterized (42–44). However, the details of these regulatory mechanisms are far from being completely understood, especially in the critical moments just after R-M system genes enter a new host cell. At this point, the host cell has a genome completely unmodified by the incoming MTase, and is thus sensitive to the incoming REase.

In this report, we focus on the C protein regulatory effects on an R-M system immediately after its introduction into a new host cell. The dynamics of gene expression relies on the C protein, which favors MTase expression initially and only later allows REase expression (45–48). In most C-associated cases, REase transcription is dependent on C-protein-mediated activation of the C gene promoter at a site called the ‘C-box’ in a positive feed-forward loop, and eventually driving expression of the downstream REase gene. The C-box structure comprises two palindromic binding sites, operator left and right, or O_L_ and O_R_, each for cooperative binding by the two C protein homodimers. O_L_ binding leads to activation, while occupancy of both sites is associated with repression. The C protein binds in a highly concentration-dependent manner, leading to efficient transcription activation at low concentrations and to repression above a certain threshold level (40,46,49). The spacing of C boxes on DNA may also affect the timing regulation, due to effects on C protein affinity (46).

**Figure 1.**
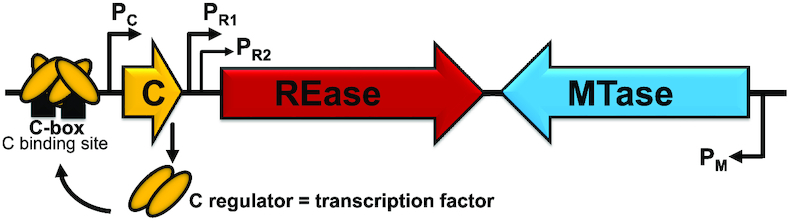
Genetic map of Csp231I R-M system (not to scale), comprising its: regulator (C gene and its promoter P_C_), REase (and its two promoters: major P_R1_ and minor P_R2_; 8nt apart) and MTase (and its P_M_). The promoters are designated by arrows. The C-box (C binding site) consists of a pair of inverted repeats CTAAG-n5-CTTAG, marked as black bars. Production of C protein results in occupation of the left part of the C-box and subsequently the left and right part as a tetramer at higher C concentrations. The C protein provides an autoregulatory negative feed-back loop for its own transcription and also, to a lesser extent, for REase transcription due to bicistronic mRNA initiated from P_C_.

The REase expression delay cannot be studied directly in most R-M system variants with a deleted C gene, because in nearly all tested C-dependent R-M systems, inactivating the C gene results in complete loss of REase expression. However, in our model Csp231I R-M system (Figure 1), REase expression is driven from a tandem array of REase promoters, P_R1_ and P_R2_, 8 nt apart, and C protein only partially affects gene expression (35). In fact, when the C promoter and the REase promoter effects on REase expression were separated, we found that the REase promoters play the dominant regulatory role. To be clear, the C protein also affects REase expression, but to a much lesser extent than in other known C-dependent systems. Csp231I C protein acts predominantly as a repressor, while other studied C proteins are more balanced between activation and repression. Strikingly, lack of the C gene—while dispensable for REase expression—greatly diminishes the efficiency of transfer to new cells and impairs the cell's fitness, as compared to WT cells (35). We hypothesized that this impairment was due to premature expression of the REase.

The aim of this study was to determine the role of C protein, as a timing regulator in the dynamic of expression of the components of the Csp231I R-M system, during its establishment in a new host cell. We used the WT Csp231I R-M system and its C-absent variant to monitor the timing of MTase and REase gene establishment in new host cells, at both the population and single cell levels. We report here, using real-time approaches to monitor expression of the newly-transferred genes, that the absence of C protein seriously affects the temporal regulation, to the point of having REase expression simultaneous with or even preceding that of the protective MTase. This effect on the host cell may be partially ameliorated by the differing diffusion behavior we observed between the MTase and REase proteins.

## MATERIALS AND METHODS

### Bacterial strains, phages and plasmids

The *Escherichia coli* K-12 strains used in this study are described below, and plasmids and phages used are listed in Table 1. Details of their construction is in Supplementary Table S1, including primers. *E. coli* DH5α*pir* was used to propagate pKD-msfGFP (50), and MG1655 Δ*rac* (51) for plasmid fluorescence, R-M system transfer assay and wherever the cross-talk of C protein and transcription factor RacR might be a biological problem (52). *Escherichia coli* JM109 served as the host strain for M13 cloning, and MG1655 [F’_ts_114lac::Tn5; Km^R^] (gift from Dr George Szatmari, University of Montreal) as the host strain for M13 phage infections. *Escherichia coli* DH5α was used for all other purposes including cloning steps.

**Table 1.** Plasmids and phages used in this study

Name	Relevant features	References
pEcoVIIIM	EcoVIII MTase gene under its natural promoter in pACYC177 vector, Km^R^. EcoVIII and Csp231I MTases have the same specificity of methylation	(35,60)
pNDL194	carrying variant of mKate2 gene of red fluorescence, pBR322 (ColE1) ori, Km^R^	(66)
pKD-sfGFP	carrying promotorless gene of monomeric superfolder GFP (msfGFP) for green fluorescence, R6K ori, Amp^R^, Requires a host strain expressing the Pir protein.	Guet lab
p18	carrying entire WT Csp231I R-M system, ColE1 ori, Tet^R^	(35)
p30	as p18, but Csp231I R-M system is devoid of C gene and its operator, but REase and MTase genes are intact, ColE1 ori, Tet^R^	(35)
p18DA	as p18, but REase of Csp231I R-M system is produced as catalytically inactive protein due to substitution of D162A of PDK motif (= R*), ColE1 ori, Tet^R^	This work
pBR::*tet*	as pBR322, but *bla* gene disrupted, Tet^R^	(35)
pHSG415	pSC101 origin, thermo-sensitive replication, Amp^R^ Cm^R^ Km^R^	(90)
pHGMCsp	as pHSG415, but MTase gene with its natural promoter cloned to disrupt the Kan resistance cassette; Amp^R^ Cm^R^	This work
pRA3	broad-host-range RA3 conjugal plasmid, the archetype of the IncU group, isolated from *Aeromonas hydrophila*, 45, 9 kb; Cm^R^ Sm^R^	(59)
ColEI::*bla*	conjugation helper plasmid, carrying the *mob* region, derivative of ColEI with *bla* gene cassette; Amp^R^	O. Werbowy, unpublished
pRKMG3	constructed in p18DA back-bone, carrying inactive full length REase (D162A) fused via GG-linker to mKate2 and active full length MTase fused via GG-linker to msfGFP. (C+ R*::mKate2; M::msfGFP)	This work
pRKMG5	as pRKMG3, but C gene is deleted, as in p30 plasmid (ΔC R*::mKate2; M::msfGFP)	This work
M13mp18	*E. coli* bacteriophage DNA vector	(91)
M13RM3	carrying the full length genes of REase (D162A) fused to mKate2 and MTase fused to msfGFP, natural C regulator is present. (C+ R*::mKate2; M::msfGFP)	This work
M13RM5	carrying the full length genes of REase (D162A) fused to mKate2 and MTase fused to msfGFP, natural C regulator is absent. (ΔC R*::mKate2; M::msfGFP)	This work
p24M-mVenus	pBAD24 derivative carrying MTase::mVenus fusion protein, Amp^R^	This work
p24R-mVenus	pBAD24 derivative carrying REase::mVenus fusion protein, Amp^R^	This work

### Fluorescence assay

To measure the fluorescence for plasmid assay, the cells were grown in LB with shaking, and culture time-point samples were collected in 20–30 min intervals during the exponential phase. Cells were gently pelleted, washed once with PBS buffer and resuspended again in 600 μl of PBS buffer. One third of each sample was used to monitor the optical density (600 nm) of bacteria, and other two thirds to read the green fluorescence (msfGFP) intensity (emission at 515 nm with an excitation at 485 nm) and red fluorescence (mKate2) intensity (emission at 633 nm with an excitation at 588 nm) in a 96-well plate reader (EnSpire Multimode; Perkin Elmer). Relative fluorescence was corrected by subtracting the level of fluorescence of non-fluorescent bacteria cells and dividing by the optical density. Arbitrary units were obtained by determining the slope of a plot of fluorescence versus culture density via linear regression. This approach both ensures that cells are in pseudo-steady-state, and provides greater precision than single-time-point assays.

### M13 infection experiments

Recombinant M13 phages were generated as listed in Supplementary Table S1, using *E. coli* JM109F’ cells, in accordance with standard methods. Prior to infection, cells were grown overnight in minimal M9-glucose medium to maintain the F′ episome, as M13 infection can select for loss of F factors (53). Infectious phage titers were determined by plaque formation on the same strain by the top agar overlay technique (54). Due to instability of M13 clones, the phage stocks were prepared from non-passaged (original preparation) M13 RF DNA via transfection.

For culture studies (bulk assays), we followed a previous report (48). Prior to infection, cells were grown overnight in LB medium and kanamycin to maintain the F’ episome. After dilution, the culture was grown in LB-medium at 30°C with shaking until cells reached OD_600 nm_ = 0.3, then split into equal portions. Of these, three were infected with M13RM3(C+), other three with M13RM5(ΔC), and one was infected with vector M13mp18 (served as negative fluorescence control). The multiplicity of infection (MOI) was 10 PFU/CFU, which is considered to sufficiently infect all cells, as tested in pilot experiments. Triplicate cultures were grown and, for each culture, samples were collected over the entire experimental time course. Preliminary infections were performed to detect the time window during which the fluorescence rises up from the background. Thus, it was established to collect samples at 5 min intervals, starting from 50 min post-infection. Samples were gently spun, resuspended in 0.85% NaCl, and kept on ice. Subsequently, the red and green fluorescence level of fusion proteins in the samples were measured at indicated parameters in a 96-well plate reader, as described in fluorescence assay. In each case, the relative fluorescence value was calculated by subtracting background fluorescence, using cells infected with vector M13mp18 as non-fluorescent control samples.

For single cell studies, infections proceeded as described above but, 20 min after infection with recombinant M13 phages, 10 μl samples of culture were placed on glass slides and covered with preheated gel pads composed of 1% agarose diluted in S7_50_ -1% glucose medium (55). Emission of fluorescence was monitored over a period of 160 min with readings taken every 5 min.

### Time-lapse fluorescence microscopy and image analysis

All images were acquired via epifluorescence microscopy, using a Zeiss Axio Observer A1 (Zeiss) with a 100× objective, immersion oil and numerical aperture (NA) of 1.45. Images were acquired using a digital EMCCD camera (Evolve, Photometrics). VisiView 2.1.4 software (Visitron Systems, Munich) was used to control image acquisition. The filters specific for red fluorophores (ET560/40x, T585lpxr, ET630/75m) to image mKate2 or green fluorescence (472/30 HC, HC BS 495, 520/35 HC) for msf-GFP detection were used. Image analyses was performed with ImageJ (FIJI package). Intensity of the fluorescent signal was determined based on the mean value of measured gray scale value with the use of segmented lines. Differences in timing for MTase and REase production were determined based on the moment at which green or red fluorescence started to be detected. The background level of fluorescence of the cells was determined by measuring the value of cells infected with vector M13mp18 (served as negative fluorescence control, same as in bulk assay). This value was then subtracted from the values observed for infected cells. The time at which the fluorescent value of observed cells rose above the background value was considered as the threshold of fluorescence detection.

### Single-molecule tracking (SMT)

The SMT setup we used is explained in (56). Briefly, the central part of a 514 nm laser beam was used for stream acquisition (20 ms integration time) of mVenus fusions, and was captured by a Hamamatsu ImageEM EMCCD camera (128 × 128 pixel area of chip used). About 160 W cm^−2^ were applied onto the image plane. Protein fusions were expressed at very low levels, then strongly excited, followed by a single step bleaching of the fluorophores. Expressing very few molecules also avoids localization artefacts due to overproduction of the respective protein. The images were taken for an average of 8 time intervals, with about 10% of tracks being longer than 10 steps. Only tracks of 5 and more steps were included in the analyses. Data analyses were done using the SMTracker 1.5 program (57,58).

### Western blot analysis

Equal volumes of culture of *E. coli* MG1655 harboring pRKMG3 or pRKMG5 plasmids were centrifuged, supernatants were removed and the cell pellets were resuspended in 40 μl of 1× SDS-PAGE Laemmli sample buffer (ThermoScientific), lysed by heating at 100°C for 10 min, and loaded onto a 12% SDS polyacrylamide gel. After separation, proteins were transferred onto a nitrocellulose membrane. Blots were analyzed with primary antibodies specific for the fluorescent proteins, IgG α-sfGFP raised in mice (ROCHE) diluted 1:4000 for detection of MTase::msfGFP and IgG α-mKate raised in rabbit (OriGene) diluted 1:40 000 for detection of REase::mKate, followed by hybridization with secondary antibodies horseradish peroxidase-conjugated, IgG α-mouse (Santa Cruz Biotechnologies) diluted 1:8000 for α-sfGFP and IgG α-rabbit (Sigma) diluted 1:30 000 for α-mKate. Visualization of bands was performed on X-ray film after addition of mixture of 1:1 luminol/enhancer and stable peroxide solutions (Thermo Scientific). The prestained MW markers used were Page Ruler (Fermentas).

### Second MTase activity-dependent cell survival assay

Cell survival was measured using the spotting assay. The overnight cultures of *E. coli* MG1655Δrac were prepared at 30°C in LB with proper antibiotics, and contained two plasmids; one carrying MTase gene on the thermo-sensitive replicon (pHGMCsp) and second plasmid with variants of R-M system: p18 (WT), p18DA, p30 or pBR::*tet* as a no R-M system control. Then they were sub-cultured, and when they reached an OD_600nm_ of 0.6 at 30°C, they were serially diluted and spotted onto three different media plates: (i) ampicillin plate incubated at 30°C; (ii) no antibiotic plate incubated at 43°C and (iii) ampicillin plate incubated at 43°C. CFU values were calculated and cell survival was determined as the ratio of CFU from ampicillin plate at 30°C divided by CFU from LB-agar at 43°C.

### Relative restriction activity assay

The restriction activity of *E. coli* cells carrying the Csp231I R-M system and its variants was measured using the efficiency of plaque formation (EOP) of phage λ*vir*. There are six Csp231I recognition sites in the λ*vir* genome. The EOP of λ*vir* was calculated as the ratio of plaques formed on *E. coli* MG1655 (or other strain) containing plasmids with no R-M system to those formed on the same strain containing a plasmid with the Csp231I R-M system or its variants.

### Conjugation mediated mobilization transfer of R-M system genes

*Escherichia coli* MG1655Δrac or *Citrobacter* sp. RFL231 (kindly supplied by MBI Fermantas, Lithuania) carrying the Csp231 R-M system were used as donors; and *E. coli* DH5α, *Citrobacter freundii* NCTC 9750 (ATCC 8090) were used as recipients. The pRA3 conjugal plasmid with streptomycin (Sm) and chloramphenicol (Cm) resistance was obtained from Prof. Grażyna Jagura-Burdzy, kindly provided by Dr Ewa Lewicka (Polish Academy of Science, Warsaw). Transformation of the *Citrobacter* cells with plasmids was performed by the standard electroporation method. The pRA3 conjugal plasmid was transferred to the donor strains carrying the Csp231 R-M system derivatives. The additional thermo-sensitive replicon plasmid pHGMCsp carrying the MTase gene in donors was used to facilitate the R-M system transfer to prepare the donors, when needed. The ColE1::*bla* plasmid (ColE1 derivative) was used as a helper plasmid for mating in conjugation dependent mobilization. Plasmid pBR::*tet* (derivative pBR322) mobilization transfer was used as a control. The method of conjugation-mediated plasmid mobilization transfer was performed as reported previously (59) with some modifications. Briefly, samples (200 μl) of the overnight cultures of the donor and recipient strains, which were grown under selection for each plasmid, were pelleted and suspended in 100 μl of fresh medium. The suspensions of recipient and donor cell were gently mixed (2:1), spread on LB-agar, and incubated overnight at 37°C or 30°C (if the thermosensitive replicon with MTase gene was used). The mixture of cells was washed away from the LB-agar, and suspended in 1 ml of LB medium. The recipient bacteria cell suspensions were serially diluted and plated on LB-agar with appropriate antibiotic, and incubated for the next 24 h at 30 or 37°C. Transconjugants were screened for their acquisition of the respective antibiotic resistance phenotypes associated with each plasmid. The obtained transconjugants were also tested to ensure relative restriction activity at the same level as the parental strain, using the bacteriophage λ*vir*; in addition the plasmids were tested by colony-PCR and analysed by restriction enzyme digestion. The mobilization frequencies were determined by dividing the number of transconjugants by the number of recipients. The limit of detection of transconjugants was estimated at 10^−9^.

## RESULTS

### Csp231I transfer to *E. coli* and maintenance there

Despite the fact the *E. coli* and *Citrobacter* are both members of the Family *Enterobacteriaceae* in the Gammaproteobacteria, Csp231I R-M system expression in *E. coli* cells without pre-methylation is lethal (35,52). For the Csp231I system to be introduced into *E. coli* cells, its genome has already to be methylated at the Csp231I target sites (60,61). We sought to test whether the additional genome protection is required for the Csp231I R-M system only during establishment (the period shortly after entry), or if it was also required for longer-term maintenance in *E. coli* cells.

**Figure 2.**
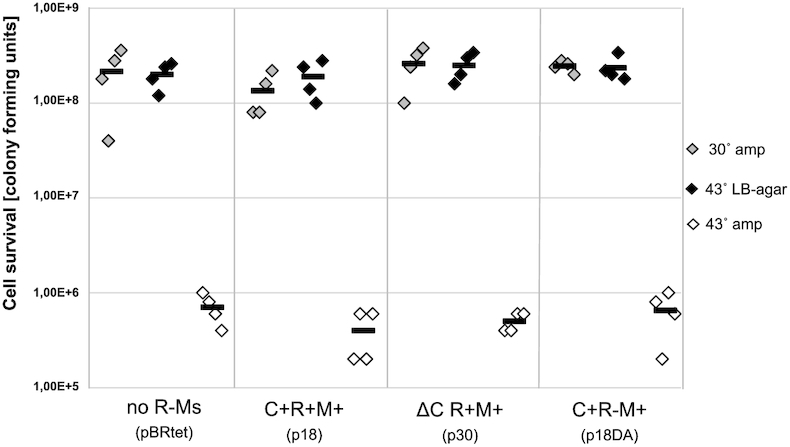
The MTase pre-expression is vital only at the stage of the Csp231I R-M system transfer into a new *E. coli* host, and C regulator does not play a role in this context. A two-plasmid system was generated. The first one was from series of plasmids harboring Csp231I R-M system variants: p18 (WT C+R+M+), p30 (ΔCR+M+) p18DA (C+R-M+), or pBRtet as a no R-M system control. The second plasmid (pHGMCsp) carried an additional, separate MTase gene on the thermo-sensitive pSC101 replicon. Cell survival after loss of the thermosensitive MTase plasmid was measured using a spotting assay and calculating CFUs. Dilutions of the cultures were spotted onto an agar plates for incubation at a permissive temperature for replication of pHGMCsp (30°C, grey dots) or at a non-permissive temperature, where MTase production is lost (43°C, black dots). To prove the MTase carrying plasmid is lost, the cell death due to lack of *bla* gene expression at 43°C on ampicillin supplemented plates (white dots) is shown. The average from four replicates is indicated by black bar.

We constructed a plasmid with the MTase gene under its natural promoter with a thermo-sensitive pSC101 replicon (plasmid pHGMCsp; Table 1). *Escherichia coli* competent cells carrying this plasmid were used for transfer of a plasmid with the intact R-M system. Next, the two-plasmid cells were cultured at the permissive temperature of 30°C. Finally, cell survival was measured after loss of the separate additional MTase gene on the thermo-sensitive plasmid during growth at 43°C. As shown in Figure 2, the survival of cells with plasmids carrying the WT R-M system and its variants (black dots *vs*. gray dots) are similar whether or not the REase gene is active. This result indicates that once WT Csp231I R-M system is established, expression of the second MTase copy is dispensable. The restriction plasmids isolated from the cells that had lost the second plasmid were shown to confer the ability to restrict bacteriophage (not shown), so this result was not due to mutation of the REase gene.

While the efficiency of Csp231I establishment in *E. coli* is highly dependent on the activity of the C protein, these experiments also showed that the dependence on genome pre-methylation was a separate phenomenon and unaffected by the C regulatory protein (compare plasmids p18 and p30 in Figure 2). These results allowed us to move forward with assessing the role of the C protein on the temporal control of the Csp231I system.

### Generating a dual-reporter system to monitor effects of the C protein regulator on R-M system temporal expression

The expectation for R-M systems entering new host cells is that REase expression will be delayed until the host's DNA is protectively methylated. This delay has been observed in other systems, but the role of C protein had to be inferred as (in those systems) eliminating the C gene abrogated REase expression (47,48). To study the temporal regulatory effects of the C protein on R-M system expression, and its hypothesized delaying effect on REase expression, we modified the already-developed M13 bacteriophage infection model (48). We used M13 phage as a vector to transfer the R-M genes, nearly simultaneously, into a population of new bacterial host cells. Such delivery mimics the natural process of R-M system dissemination via horizontal gene transfer and, significantly, can be done using physiologically normal growing cells, without the harsh treatments needed to make *E. coli* competent. Moreover, this exchange likely resembles the normal genetic flux between *Enterobacteriaceae* members (such as *E. coli* and *Citrobacter)*, which naturally exchange their genetic material via conjugation/mobilization and transduction (62). M13 phage is a temperate phage, and infected cells are not lysed – their growth is slowed, so one can observe pseudoplaques on lawns, but phage progeny are continuously extruded across the cell wall of living cells (63,64). Infection is usually very efficient, relatively synchronous and the phage DNA quickly penetrates the host cells making the system optimal for real-time monitoring of expression of newly-introduced genes (48).

**Figure 3.**
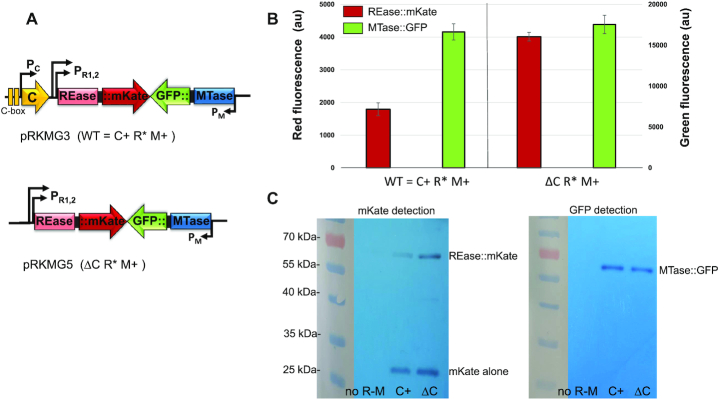
Steady-state expression of R-M system fusion proteins. (**A**) Generated constructs contain entire ORF length fused to fluorescent reporter genes, with or without C regulatory protein, as shown. C protein binding sites (C-boxes) are indicated. In both cases, the inactive REase (R* = D162A, substitution in conserved catalytic center) is produced as a fusion to mKate, while the active MTase is expressed as a MTase::sfGFP fusion. (**B**) The level of expression for fusion proteins REase::mKate and MTase::sfGFP for R-M systems with (C+) and without C protein (ΔC) is measured in relative fluorescence (red and green) arbitrary units on separate y axes. (**C**) Production of fusion proteins was confirmed using commercial antibodies against fluorescent proteins on cell extracts from *E. coli* carrying pRKMG3 (WT C+) or pRKMG5 (ΔC). Cell extract without plasmid was used as negative control (no R-M). Expected MW: REase::mKate - 63.9 kDa, MTase::sfGFP - 61.2 kDa, mKate alone - 26 kDa.

We first generated in-frame fusions of the MTase and REase genes with two different fluorescent reporter proteins (Figure 3A). The entire operon for the Csp231I R-M system (WT and its C-deleted variant) was cloned into a pBR322 vector plasmid. We used the inactive REase variant (D162A), which differs only by one amino-acid at the conserved catalytic core of a PD-(D/E)XK nuclease motif (65). In this way we could avoid cell killing during M13 infection, without the need to complicate our system with an additional MTase plasmid. The fused MTase::msfGFP exerted its full activity, as assessed by phage protection assays (Supplementary Figure S1). We consider this version of the Csp231I system, which includes its natural regulators and native expression sequences, as being ‘WT’. The full-length REase and MTase genes were next each fused in-frame, with a –GlyGly– linker, to the reporter genes coding for the fluorescent proteins mKate (red, REase) and msfGFP (green, MTase). In both cases, the fluorescence signal is driven by the natural transcriptional and translational signals from the REase and MTase genes, and the reporter genes are promoterless.

These reporters were chosen based on their excitation with different fluorescence filter sets. The red reporter, mKate2, is modified to be much brighter and more bleaching resistant than mCherry (66), and the green reporter is a monomeric super folding variant of GFP (msfGFP). Finally, two different constructs, one carrying the sequence coding for C regulatory protein (pRKMG3; WT = C+) and one without the C gene (pRKMG5; ΔC), were created (Figure 3A). The production of correct fusion proteins was confirmed by both reporter assays (Figure 3B) and western blot analysis (Figure 3C).

The levels of expression of REase::mKate and MTase::sfGFP, in the presence (C+) and absence (ΔC) of C protein, were measured. The results indicate that lack of C protein stimulated REase expression almost two-fold (Figure 3B), which is in agreement with previous studies showing higher (∼3-fold) phage restriction for R-M system with the C gene deleted (plasmid p30) (35). Again, this differs from the effects of C protein in some other R-M systems, in which C is both a repressor and an activator, depending on its concentration (37,38,45). In our assays, the MTase level stays the same regardless of C protein presence or absence. Western-blot analysis of lysates from cells carrying plasmids with fluorescently labeled REase and MTase, using the specific antibodies against mKate2 and sfGFP, revealed the predicted band sizes with undetectable degradation. However, the cell lysates for REase::mKate also revealed the presence of a certain amount of free mKate expression (Figure 3C). As will be described below, this resulted from an adventitious translation initiator at the Gly–Gly junction with the mKate reading frame, but expression was nevertheless still dependent on transcription from the REase promoter.

### Population-level measurement of C protein effects on the temporal dynamics of REase and MTase gene expression

**Figure 4.**
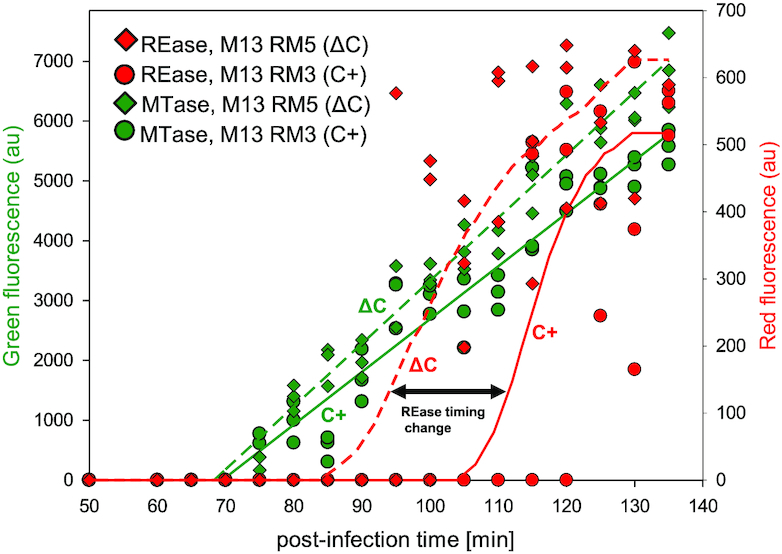
*In vivo* kinetics of Csp231I restriction-modification gene expression after entering a new host cell. The R-M system was delivered to host cells by recombinant M13 phages during infection as described in Material and Methods section. The fluorescence signals were separately monitored in cultures in 5 min intervals, up to 140 min post-infection, to detect expression of REase::mKate (red) and MTase::sfGFP (green) in biological triplicates. The relative fluorescence (red and green) was measured in arbitrary units and shown on separate y axes. To compare the effects of C regulatory protein on R-M system transfer, the host cells were infected with recombinant M13 phages either carrying the C gene (M13RM3, C+, circles) or without the C gene (M13RM5, ΔC, diamonds). The trends for MTase expression (green) and REase (red) are shown by continuous (C-present R-M system) or dashed (C-absent R-M system) lines. The 15 min−shift in time for REase expression (C+ versus ΔC) is indicated by the black double arrow

To study Csp231I R-M gene establishment in a new host, the fused genes for fluorescently labelled REase and MTase (Figure 3A) were cloned into M13 replicative form DNA (M13mp18), and two different stocks of recombinant M13 phages were generated: M13RM3 (WT C+) and M13RM5 (ΔC) (Table 1). In a series of pilot studies, the stocks were confirmed to infect cells, yielding the same expression patterns as had been seen with the steady-state R-M system of plasmid origin (Supplementary Figure S2). Next, the *E. coli* MG1655 F’ host cells were grown in LB liquid medium into exponential phase, and then split into equal portions (Materials and Methods). Cultures were infected with recombinant M13 phages carrying the R-M system with (M13RM3 WT C+) or without (M13RM5 ΔC) the C regulator, at an MOI of 10 (10PFU/1CFU) to ensure a low background of uninfected cells. The culture samples were collected over a period of 140 min after M13 infection, at intervals of 5 min, and they were used to measure the level of fluorescent MTase (green) and REase (red) in a bulk assay (Figure 4). The real-time R-M system establishment data indicate that MTase expression appears at about the same time post-infection, regardless of the presence or absence of the regulatory C protein. Specifically, the green fluorescence signal rises above the baseline in comparable linear trends, starting about 70 min post infection (Figure 4, green diamonds *vs*. green circles).

In addition, production of MTase precedes production of REase (red) whether or not C protein is present. However, with the WT R-M system the REase appears about 35 min after the MTase, whereas in the absence of C protein the delay in appearance of REase is reduced by more than half, to about 15 min (Figure 4, red diamonds *vs*. green circles). Moreover, in the absence of C the REase expression reveals a rather sharp increase from background, as compared to MTase appearance. These results clearly confirm the role of C protein as a temporal regulator of REase gene expression.

### Single cell analysis reveals greatly decreased delay in REase expression in the absence of the C regulator

**Figure 5.**
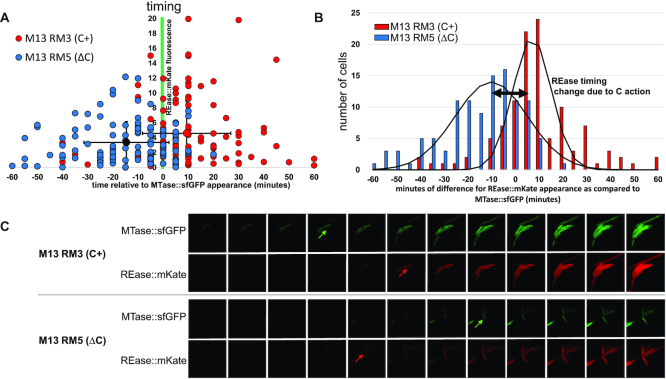
REase delay is disturbed in cells lacking the C regulator as monitored in real-time at the single cell level. (**A**) Timing of REase expression (red fluorescence) in individual host cells, infected by recombinant M13 phages carrying R-M system with C protein (red points; N = 111) and without C protein (blue points; *N* = 110). The times are set in reference to appearance of MTase expression (green fluorescence detection), defined as time 0 (x axis). The vertical axis indicates the intensity of rising red fluorescence, which crossed the background red fluorescence baseline at the earliest time. The average time values for REase expression for both +C and ΔC systems are indicated with error bars (standard deviations), which are each about ±15 min. Mean REase expression timing for the ΔC R-M system is ∼15 min earlier than MTase expression, and about 10 min later than MTase expression for the +C R-M system. The two-tailed P value between the ±C groups is <0.0001. (**B**) Distribution curve of scattered points from panel A, showing the number of cells for each variant (ΔC versus +C) grouped by timing values of appearance of red fluorescence (REase expression) in comparison to appearance of green fluorescence (MTase). The black lines represent the trend lines indicating a roughly normal distribution for both variants. The double-headed arrow indicates the shift in means, of ∼15 min. (**C**) Representative series of time-lapse images taken independently for cells infected with the two variants of recombinant M13 phages (+C versus ΔC). The 12 frames cover a 60 min time range, with each shot taken at 5 min intervals after M13 infection. Arrows indicate the time of detection of rising fluorescence from no fluorescent background, red fluorescence for REase and green for MTase. In the upper panel for the +C- R-M system, REase expression is detected 10 min after MTase, whereas in the bottom panel for the ΔC R-M system, the REase expression precedes the MTase detection by about 15 min.

A major goal of this work was to visualize C-protein-dependent changes in gene expression at a single cell level. Thus, the same experiment as in the previous section was performed at the individual cell level. The recombinant M13 infections were monitored over a period of 160 min post-infection, *via* time-lapse epifluorescence microscopy at single-cell resolution. Pictures were taken at intervals of 5 min, using filters specific for red or green fluorescence, for mKate and sfGFP detection respectively. About 220 time-lapse images were analyzed to determine the difference in timing for MTase and REase production after entry to the new host (Figure 5). Due to the transfer of M13 infected cells onto microscopy slides, which necessarily involved some change in growth conditions, we could not refer to the level of fluorescence in exact minutes post-infection. Rather, we measured the relative earliest times of appearance of red and green fluorescence signals in each individual cell. Therefore, the moment in which the green fluorescence (MTase) has started to be detected was established as reference time 0 and the difference in time of production of REase was set in comparison to MTase expression (Figure 5AB).

The results show that in cells receiving the WT R-M system (WT C+), production of REase appears only with a stable time delay averaging about 10 min after the MTase. The delay between MTase and REase was observed for a majority of cells within the range of up to 20 min of REase expression after MTase (Figure 5A, red circles; Figure 5C). The cell fraction, where the REase was detected at or later than the time of MTase appearance was 82.7% (91 out of 110 monitored cells). In contrast, for cells receiving the R-M system variant without regulatory protein (ΔC), REase production occurred substantially earlier than for the WT R-M system. More importantly, the (catalytically inactive) REase appears often at the same time or even preceding production of MTase, with an average of 15 min before MTase (i.e. difference of 15 min; Figure 5A, blue circles; Figure 5C). The cell fraction, where the REase was detected at the time of MTase appearance or earlier was 84.7% (94 out of 111 monitored cells). The two variants (C+ versus ΔC) each yielded a normal distribution in MTase-REase time difference, and the respective means confirmed the significant REase timing shift (Figure 5B).

Because there was a limited amount of free (unfused) mKate protein made (Figure 3C, described above), we also tested whether the free mKate might affect our interpretation of the relative timing of REase-expression. We altered the recombinant M13 phages M13RM3 (C+) and M13RM5 (ΔC), introducing a frameshift mutation into the REase gene. This would result in all of the mKate fluorescence signal coming from translation starting at the adventitious initiator in the linker (Supplementary Figure S3). Infection with the REase-frameshifted recombinant M13 showed first detection of red fluorescence much later than the time range measured for the non-frameshifted M13RM3 and M13RM5. Specifically, with REase appeared about 67 min after MTase for the C+ variant (compared to 10 min after for the fused construct), and 20 min after MTase for the ΔC variant (compared to 15 min before the MTase for the fused construct). This demonstrates that, while the unfused mKate protein may have affected the total amount of REase expression inferred, it had no effect at all on the measurement of expression timing relative to the MTase.

### Single molecule tracking of REase and MTase action

The optimal temporal expression of an R-M system depends on how it functions in the host cell. We thus wished to gain further insight into the mode of action of REase and MTase *in vivo*. We employed single molecule tracking (SMT) to investigate how REase and MTase might find their target sites on the bacterial chromosomes, respectively. We employed YFP-based SMT (56) and expressed REase and MTase-mVenus fusions (mVenus is a brighter variant of YFP; see Table 1 and Supplementary Figure S4 for corresponding western blot analysis). The fusions were expressed from plasmids using extremely low induction levels, such that single fluorophores are obtained almost instantaneously, after few initial frames in which molecules bleach, and then can be tracked in real time.

**Figure 6.**
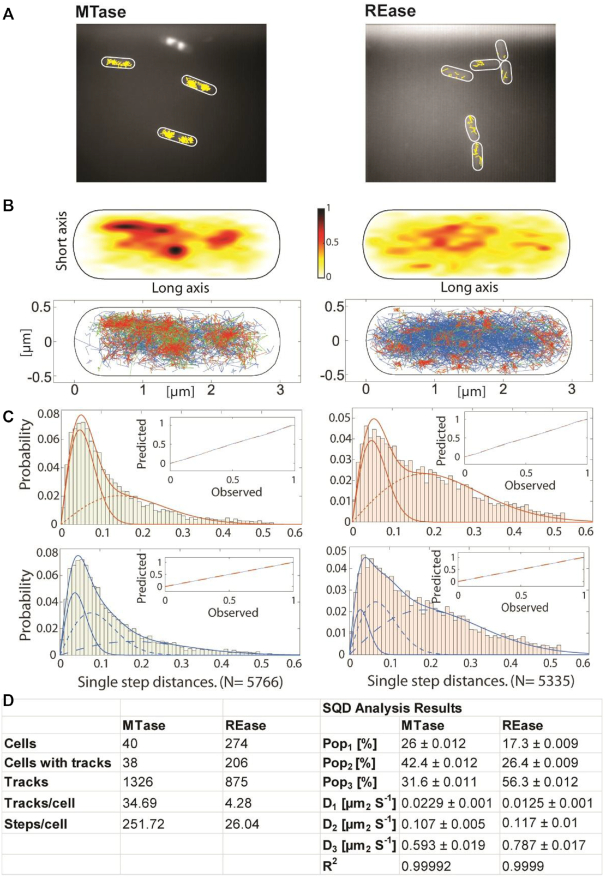
Single molecule tracking analyses of the MTase and REase, expressed at very low levels as mVenus fusions. (**A**) Representative tracks (in yellow) of enzyme molecules, in cells that are outlined by white ovals. (**B**) Projection of all tracks into a standardized cell of 3×1 μm size. Upper panels are heat maps, where darker shading indicates higher presence of molecule tracks. Lower panels are confinement maps, with tracks: (i) moving within a radius of 120 nm for at least five steps are shown in red, (ii) tracks moving freely in blue and (iii) tracks containing both confined and free motion in green (‘transitions’). (**C**) Jump distance diagrams showing the cumulative probability distribution of squared displacement analyses (SQD) to estimate the diffusion constants (D) and relative fractions of up to three diffusive states. Upper panels show data fitted with two Rayleigh distributions, lower panels with three distributions. Inset show deviation (blue line) of experimental data from modelled data (indicated by dashed red line). (**D**) Summary of data obtained from SQD analyses, diffusion constants D_1–3_ correspond to populations Pop_1–3_.

We analyzed the obtained tracks within cells (1326 for MTase, 875 for REase, Figure 6AB), and projected them into a medium-sized cell of 3×1 μm size (heat map, Figure 6B, upper panels). It became clear that movement of the MTase is largely restricted to nucleoid-containing spaces of the cell, while the REase tracks are more randomly scattered throughout the entire cell (Figure 6A, B). In addition, we performed a molecule jump distance plot from squared displacement analyses (SQD), using a Rayleigh fitting procedure (Figure 6C, upper panels). Observed data could be best explained using three fits, indicating that assuming three distinct populations can explain our data (Figure 6C, lower panels). One population comprised slowly-diffusing molecules showing little displacement (low diffusion constant, D_1_, Figure 6D), likely consisting of molecules bound to the DNA and engaged in enzymatic activity, or at least stabilized by base flipping (67). A second population showing intermediate step sizes could comprise the molecules interacting with DNA non-specifically (68,69) and thus moving through the nucleoids in a constrained manner (D_2_ at Figure 6D). The third, most dynamic population likely consists of freely diffusing molecules (highest diffusion constant, D_3_ in Figure 6D). Molecules were tracked with an integration time of 20 ms, such that calculated diffusion constants for freely diffusive molecules are underestimates, because molecules that show considerable movement within 20 ms are not captured as a single point spread function (Figure 6D). Fitting with three Rayleigh distributions yielded an R squared value of almost ‘1’, suggesting that assuming four populations would result in overfitting of the data (Figure 6D). Due to the apparent presence of three observed molecule populations, their action scenarios might be outlined as just described: specific DNA binding, non-specific DNA interactions and free diffusion. Using the SMTracker 1.5 program (57,58) we determined that 26% of MTase molecules were present in a static (stably DNA-bound) state, while 42% moved in a constrained manner, and about 32% in a freely diffusive manner.

In contrast to the MTase, only 17% of REase molecules were observed in a slow-mobile/DNA bound state, and only 26% in a medium-mobile state, while a majority of 56% were freely diffusive. These data suggest different modes of movement and target searching for the MTase and REase. One of the parameters describing this contrast in the enzymes’ motion is their confinement, defined as a molecule pause within a radius of 120 nm (4 times the localization error) for at least 9 consecutive steps. We analyzed the confinement maps of MTase and REase and divided their tracks into groups: showing little displacement in time (confined motion), freely diffusive (non-confined motion) and those switching between both states. The results indicate that MTase is largely engaged in confined motion and DNA-bound states, suggesting it predominantly slides along DNA to find its sites of activity. In contrast, REase finds its targets more directly from a diffusive state, and seems to employ DNA-sliding to a much lower degree, if at all. The confined motion of MTase is largely restricted to the cell center, and likewise transition events, whereas REase arrests at various positions within the cells (Figure 6B, bottom panels). The most intensive sites of confined motion appear to occur towards the cell center (i.e. in the nucleoids), where DNA targets are present. In any event, these analyses reveal strikingly different modes of diffusion through the cell for REase and MTase and support the idea that MTase employs 2D DNA sliding as a major mechanism for target search, while REase engages predominantly in a diffusion-capture mechanism for finding its DNA binding sites. We discuss the implications of this below.

### Lack of C regulator affects the transfer of Csp231I R-M system during plasmid mobilization

Our observation from Figures 4 and 5 that lack of C regulator substantially reduces the delay in REase expression may have serious consequences for the R-M system horizontal transfer between the cells. We asked whether we could observe the predicted difference in efficiency of R-M system transfer where the REase is active, but with or without C protein.

We established a quantitative system of conjugative plasmid mobilization, in which a given R-M system plasmid enters new host cells lacking pre-methylation at that R-M system's specific sites. Further, to make the system as similar as possible to naturally-occurring R-M system transfer, the recipients carried some other R-M systems. First, we confirmed that the broad-host-range pRA3 conjugal plasmid could be transferred from *E. coli* to *Citrobacter* and *vice versa*, as well as within the same species, with high frequencies of trans-conjugants per recipient cell (from 7 × 10^−1^ up to almost 1; not shown). In addition, we used a conjugative helper plasmid (ColE1::*bla*) that carries the *mob* region, to support the mobilization transfer of *mob*-deficient plasmids in all selected hosts (70,71). Under our conditions, ColE1::*bla* and pBR::*tet* were both present in all trans-conjugants confirming the relatively high efficiency of mobilization by the pRA3 plasmid (not shown).

We began by testing the route of R-M system transfer between the two *E. coli* laboratory strains: MG1655 and DH5α (Table 2, upper part). Comparing the two REase+ strains, ±C protein, the WT R-M system (p18 plasmid, with active C protein and active REase) transfers at ∼4.5× higher frequency than when the C protein is absent (p30; R+C–). Comparing the two C+ strains, ±REase activity, the catalytic activity of the REase had a small effect (WT p18 versus p18DA; R+/R– = 0.4).

**Table 2.** The pRA3-mediated transfer frequency of mobilizable plasmids carrying the R-M system in different mating pairs

		Recipient	
		*Escherichia coli*	*Citrobacter* sp.
**Donor**	***E. coli***	MG1655Δ*rac* [pRA3, ColE1::*bla*] × DH5αRif	MG1655Δ*rac* [pRA3, ColE1::*bla*] × NCTC9750 [pACYC177]
		WT R+C+ = 5.8 (±1.9) ×10^–7^	WT R+C+ = 5.84 (±0.64) ×10^–7^
		R+C- = 1.3 (±0.4) ×10^–7^	R+C- = 3.20 (±0.37) ×10^–7^
		R-C+ = 1.4 (±0.8) ×10^–6^	R-C+ = 6.65 (±2.23) ×10^–6^
		V R-C- = 4.2 (±0.1) ×10^–2^	V R-C- = 3.15 (±1.93) ×10^–2^
		**C+ / C- = 4.5**±2.0 *	**C+ / C- = 1.8**±0.3*
		**R+ / R- = 0.4**±0.3	**R+ / R- = 0.09**±0.03*
	***Citrobacter***	Csp sp.RFL231 [pRA3, ColE1::*bla*] × DH5αRif	Csp sp.RFL231 [pRA3, ColE1::*bla*] × NCTC9750 [pACYC177]
		WT R+C+ = 1.4 (±0.9) ×10^–5^	WT R+C+ = 2.44 (±1.13) ×10^–6^
		R+C- = 9.3 (±1.2) ×10^–7^	R+C- = 3.76 (±1.35) ×10^–7^
		R-C+ = 1.4 (±0.7) ×10^–5^	R-C+ = 2.76 (±0.82) ×10^–6^
		V R-C- = 2.1 (±0.5) ×10^–2^	V R-C- = 1.63 (±1.12) ×10^–2^
		**C+ / C- = 15**±10	**C+ / C- = 7**±4
		**R+ / R- = 1**±0.8	**R+ / R- = 0.9**±0.5

The calculations are shown as the number of obtained transconjugants per recipient cell. The mobilized plasmid genotypes are as follows: WT R+C+ (p18); R+C- (p30); R-C+ (p18DA); Vector (V) R-C- (pBR::*tet*). R – restriction; C – regulatory protein. The donor *Citrobacter* sp. RFL231 is the original strain, carrying the Csp231 R-M system on its genome, whereas the recipient - *Citrobacter* sp. NCTC9750 has no methylation for the specified Csp231I R-M system. The effects of C regulatory presence and restriction activity is shown as a rate of transfer frequency for appropriate pair of plasmid derivatives. Shown are mean values for three independent measurements, and the standard deviations. Statistical analysis indicate these differences are considered to be statistically significant (∗). To assess statistical significance, two tailed Student's unpaired t test was used with GraphPad Prism software (GraphPad Software), with a *P* value cutoff of <0.10 (90% confidence interval).

Similar trends were observed when an *E. coli* donor was mated with *Citrobacter freundii* as the recipient. However, the C regulator effect was about half what had been seen in the *E*.*coli - E. coli* transfers, with about 2-fold better mobilization for C+ R-M system plasmids than when C protein was absent. In this strain pair, however, the presence of functional REase reduced successful plasmid transfer by nearly an order of magnitude.

Next, we sought to test the same plasmid transfer, but with transmission from a natural host - *Citrobacter* sp. RFL231 (Table 2, lower part). Regardless of the recipient species, *E. coli* or *Citrobacter*, the C+ R-M system variants were transferred at much higher efficiency than C- variants - almost 15-fold for *E. coli* and 7-fold for *Citrobacter*. However, in both cases, the recipients having unmethylated genomic DNA are tolerant of the active REase during establishment, as there is no difference in the transfer ratio of constructs with and without REase catalytic activity. Thus introduction into *E. coli* of the WT Csp231I R-M system *via* conjugation, in contrast to transformation, does not required pre-methylation of the new host.

Finally, we screened transconjugants, and confirmed that they are phenotypically identical to the parental donors, and that the MTase and REase are active. We also observed very high frequency of transfer for the control pBR322-derived plasmid as compared to other same-vector plasmids, but this feature may be associated with its smaller size.

## DISCUSSION

R-M system operons use complex intertwined layers of regulation, including temporal dynamics, to successfully spread through the bacterial world (28). One of the strategies is to engage simple genetic timing circuits to facilitate their establishment following horizontal gene transfer. We have experimentally determined the temporal pattern of transfer of an interesting type of R-M system, revealing an essential function for a regulatory protein in separating the necessary timing of expression of the two R-M catalytic genes (MTase and REase). To date, the primary timing role of the C regulatory protein has been directly documented for only two R-M systems: PvuII and Esp1396I (47,48). Both studies showed the key role of the C protein in mediating a delay in REase expression, enabling the MTase to sufficiently complete the modification process of the new host's genome, which is essential for successful R-M system mobility, and necessary for post-segregational killing (47,48,72,73). However, the previous studies, though both elegant and informative, could not directly demonstrate the C protein effect, since in PvuII and Esp1396I there is no REase expression in the absence of C protein. Thus the key control using a C gene-deleted R-M system to assess expression timing could not be performed. Those experiments demonstrated a timing difference between appearance of MTase and that of REase, but could not provide direct determination of a causal role of the C protein in the dynamics of REase expression. Here, we exploited the unique properties of the Csp231I R-M system, allowing such studies. Specifically, due to the presence of separate tandem promoters for the REase gene, REase expression is substantial even in absence of C protein (35).

In this report, we used a previously-tested genetic system, with M13 phage as the vehicle delivering the R-M system operon, though in our case with the REase and MTase genes fused to fluorescent proteins. The M13-based system has advantages over other possible means of transfer, such as transformation or conjugation (48), including relatively simultaneous gene introduction and lack of physiologically disruptive treatments. The real-time monitoring of individual live cells enabled us to determine, relatively precisely, the expression of transfected DNA, in basic agreement with previous population-level studies (74,75). Our results showed that the delay between MTase and REase expression for WT Csp231I R-M system is about 35 min, as compared to PvuII R-M system in the same phage delivery system in cell culture, which was about 10 min. However the PvuII values were determined based on mRNA levels, not the detection of activity of gene products. Similarly, different host and culture conditions might slightly affect the delay values.

In another report that measured the MTase-REase delay at the single cell level, it took between 90 and 350 min to detect REase after MTase appearance (47). This relatively long delay was about an order of magnitude greater than the ∼20 min delay seen in our data at single cell level. This difference might be reflected by several factors. First, the Esp1396I R-M system used in the mentioned studies was carried by the very high copy number vector pUC19, which resulted in very high expression of tested genes, while under natural conditions the operon is carried by a low-copy *Enterobacter* plasmid (76). Second, the overexpression might explain why the Esp1396I R-M system acceptor cells in that study showed a DNA damage phenotype, manifested by abnormal, filamentous cell morphology, possibly due to excess REase production. Under conditions with an active SOS response, cells cannot grow with normal generation times and have substantially-disturbed patterns of gene expression (77,78), perhaps contributing to this timing.

The M13 phage system allows the fairly synchronous introduction of genes into the new cells, with much less physiological disturbance. In addition, *E. coli* cells do not undergo natural transformation, so the artificial transfer of genes following harsh steps, such as cold/heat shock, cell washing, and an immediate shift into a rich medium, might substantially affect expression of the newly-introduced genes. The MTase/REase relative expression could thus be observed, but the delay values for Esp1396I R-M system are relatively scattered (47). In addition, as with PvuII, the kinetic studies on Esp1396I did not result the direct determination of the C protein effect on REase expression, as only the WT (REase+) system could be monitored.

Our study demonstrates that C function delays the timing of REase appearance, giving the MTase time to complete its host genome methylation. As noted above, specific features of Csp231I allowed us to determine the role of C protein directly. In our case, the delay in REase expression was reduced by about 20 min in the absence of C protein, as measured in cell culture studies (Figure 4). In fact in single-cell studies, in the absence of C protein the delay was eliminated altogether and REase and MTase appeared almost simultaneously (Figure 5). A theoretical study based on simulations of genetic circuits reveals that operons with an autogenous regulatory protein are predicted to result in a relatively short delay, compared to circuits not controlled by an autogenous regulator (79). Perhaps, the fusion of the DNA segment carrying C gene to segment with REase gene with its own promoters, as exemplified in Csp231I R-M system, benefits the R-M system function and its dissemination, providing an example of regulatory element as a solution of an evolutionary optimization problem (80).

C-mediated timing delay would seem to be essential to limiting the recipient's genome exposure to REase cleavage, and thus to increasing host cell fitness (35,81). The findings of our investigation of an active C-deficient R-M system transfer to new unmethylated host cells (Table 2) can have different explanations. Conjugational transfer showed that the C-present R-M system is accepted more readily than C-absent, in all tested mating pairs. However, the restriction-negative controls indicated that only the transfer of active R-M genes from *E. coli* into *Citrobacter* are affected by the active REase due to lack of C protein. In the other three conjugal mating pairs, the active REase seems not to reduce transconjugation efficiency. This effect might be dependent on the conjugational system used. For example, the transfer of the active EcoVIII R-M system, mediated by an F conjugal plasmid, resulted in a strong toxic effect when *Citrobacter freundii* was used as a donor and *Citrobacter freundii* or *E. coli* HB101 used as recipients (62). However, the same transfer mediated by plasmid R64drd11 did not show any difference between restriction-positive and negative variants (unpublished data). The genetic bases of this phenomenon are not clear. In addition, the effects of C protein gene transfer between two *E. coli* strains (Table 2) might be explained in part by broader regulatory effects of C protein itself (52).

Based on the differential timing of MTase and REase expression, we were curious to determine whether the two enzymes use similar or distinct strategies to find their target sites on the genome. Single molecule experiments revealed a predominant movement of MTase molecules within central spaces in the cells, corresponding to the nucleoid. About a quarter of the molecules were moving in a confined space, likely representing specifically DNA-bound species. Nearly twice that number were moving with an intermediate diffusion coefficient, similar to that found for SMC (structural maintenance of chromosomes) protein, a sequence-nonspecific chromosome compaction factor that moves through the nucleoid in a manner of constrained diffusion (82). Our observations on MTase dynamics on DNA supports its having a more processive than distributive action (at least more processive than REase), similar to the *E. coli* Dam MTase, which methylates several target sites on DNA without dissociation (83). Usually, the MTases of Type II R-M systems (such as EcoRI MTase) have a distributive mode of action (84), however we cannot definitively determine the processivity of Csp231I MTase without further biochemical studies.

In contrast, a majority of Csp231I REase molecules were determined to be in a state with the highest diffusion coefficient, *i.e*. likely free diffusion. This suggests that this REase does not preferentially move within the nucleoid like MTase. The EcoRV REase was found to rapidly locate a recognition site within long, non-cognate DNA in a process of facilitated diffusion (85–87). Another REase, EcoRI, slides upon leaving a site (so more processive), while R.EcoRV appears to leave the DNA (hopping; more distributive) (88). It seems that REases may use much less DNA-based constrained motion, but more a diffusion/capture mechanism for targeting site specific hydrolysis of DNA strands.

It is tempting to speculate that target search for MTase is more efficient based on the higher degree of nucleoid engagement than the more stochastic-appearing target search for REase, where the REase diffuses throughout the cytoplasm. One might predict this difference in behavior for a system in which the role of the MTase is to maintain nucleoid methylation, while that of the REase is to contact DNA entering the cell from the outside, at its periphery. A report on direct biochemical studies on competition on DNA between MTase and REase of the same Type II indicated that MTase is slightly more efficient than the cognate REase at specific site location and catalysis (84). Thus, in general, the MTase/REase enzyme target searching features alone might generate a lag, favoring the MTase to act more quickly, which could be further modulated by unequal enzyme amounts associated with the timing of their expression. This difference in MTase *vs*. REase diffusion may reduce, while not eliminating, the criticality of the C protein's activity during R-M system establishment.

Based on a growing body of research, it is fair to say that the ‘inter-species journey’ of intact R-M systems is a challenge. Potential problems include insufficient MTase expression, MTase incompatibility with Type IV systems such as McrBC (16), overexpression of the REase, and insufficient repair capabilities (89). Another potential problem, addressed here, is the timing of REase expression. After entering a new host, there is likely strong selection to optimize the temporal control system. Our knowledge of significant gene-transfer processes is still fragmentary, and their molecular bases over time and across subcellular space are yet to be elucidated.

## Supplementary Material

gkab183_Supplemental_File

## Notes

Present address: Alessandro Negri, Laboratory of Molecular Bacteriology, Intercollegiate Faculty of Biotechnology UG-MUG, Medical University of Gdańsk, Gdansk, Poland.
