# Supplementary material for: Regulator-dependent temporal dynamics of a restriction-modification system's gene expression upon entering new host cells: single-cell and population studies
Source: Nucleic Acids Res. 2021 Mar 21;49(7):3826–40. doi: 10.1093/nar/gkab183 (PMC8053105; doi:10.1093/nar/gkab183)

Regulator-dependent temporal dynamics of a restriction-modification system's gene expression upon entering new host cells: single-cell and population studies

Supplementary Materials:

Alessandro Negri[1#], Olesia Werbowy[1], Ewa Wons[1], Simon Dersch[2,3], Rebecca Hinrichs[2,3], Peter Graumann[2,3] and Iwona Mruk[1*]

1 Department of Microbiology, Faculty of Biology, University of Gdansk, Wita Stwosza 59, Gdansk 80-308, Poland
2 SYNMIKRO, LOEWE Center for Synthetic Microbiology, Marburg, Germany
3 Department of Chemistry, Philipps Universität Marburg, Hans-Meerwein-Strasse 6, 35032 Marburg, Germany

# present address: Laboratory of Molecular Bacteriology, Intercollegiate Faculty of Biotechnology UG-MUG, Medical University of Gdańsk, Gdansk, Poland.

*Corresponding author

Address for correspondence:

Dr. Iwona Mruk, Department of Microbiology, University of Gdansk, Wita Stwosza 59, Gdansk 80-308, Poland

e-mail: shamrock127@hotmail.com

Table S1. Supplementary plasmids, strains and primers list

M13mp18	Replicative form of phage M13. P_{lac}, *lacZ, lacI*.	Yanisch-Perron et al., 1985
p18	carrying entire WT Csp231I R-M system, pBR ori, Tet^R	C+<br>R<br>M<br>Tet^R<br>Rezulak et al., 2016
pKate20	Based on p18. R.Csp231I and M.Csp231I have been cut by BstBI and StuI, resulting in only a 5'-end fragment of the genes. The sequence encoding mKate, amplified from mutagenised pNDL194 (using truekate1 and RevKateR primers), has been inserted using BstBI and StuI cut site, resulting in fusion gene of fragment of R.Csp231I and entire mKate connected by a GG linker.	C+<br>R'::mKate<br>M'<br>Tet^R<br>This work
pMFG23	Based on pKD3 carrying gene for sfGFP. The sequence coding for entire M.Csp231I preceded by promoter (amplified from p18 using pFMpKD3 and pRMFULLpKD3 primers) has been inserted using NsiI cut site upstream of sfGFP, leading to the creation of the fusion gene of entire M.Csp231I and sfGFP connected by a GG linker.	M::sfGFP<br>Amp^R<br>This work
pRKMG2	Based on pKate20. Leftover of fragment M.Csp231I has been removed by cut with PstI and StuI and replaced by fusion gene of M.Csp231I and sfGFP from pMFG23 (amplified using PFMFG1 and PRMFG2 primers and cleaved with PstI and HincII).	C+<br>R'::mKate<br>M::sfGFP<br>Tet^R<br>This work
pRKMG3	Based on pRKMG2. Addition of the fragment (amplified from p18 using PF18Rfull1 and PR18Rfull2 primers) to obtain full sequence of R.Csp231I within BstBI cut site. Fragment was mutagenized to change the codon in position 162 from D to A in order to inactivate the restriction activity (using PF18mutR1 and PR18mutR2 primers). Resulting in fusion of the full sequence of R.Csp231I and mKate connected by a GG linker.	C+<br>R(D162A)::mKate<br>M::sfGFP<br>Tet^R<br>This work
pRKMG5	Based on pRKMG3. It presents deletion of the sequence coding for C.Csp231I. Deletion has been performed by restriction with EcoRI, which resulted in removal of C sequence and partial R sequence. Entire R sequence was restored introducing the missing R fragment (amplified from p18 using C23 and qR-REV primers) within EcoRI cut site.	ΔC<br>R(D162A)::mKate<br>M::sfGFP<br>Tet^R<br>This work
M13 RM3	Based on M13mp18. The sequence coding for the entire Restriction-modification system comprising the fluorescent proteins from pRKMG3 (amplified using PFMFG1 and PRC2 primers) has been introduced within the *lacZ* gene using PstI and BamHI cut site. Infected cells do not produce β-galactosidase	Phage M13<br>C+<br>R(D162A)::mKate<br>M::sfGFP<br>No antibiotic resistance<br>This work
M13 RM5	Based on M13mp18. The sequence coding for the entire Restriction-modification system comprising the fluorescent proteins from pRKMG5 (amplified using PFMFG1 and PRC2 primers) has been introduced within the *lacZ* gene using PstI and BamHI cut sites. Infected cells do not produce β-galactosidase	Phage M13<br>ΔC<br>R(D162A)::mKate<br>M::sfGFP<br>No antibiotic resistance<br>This work
p24M-mVenus	Based on p24MG2 (pBAD24 derivative carrying the sequence coding M.Csp231I in fusion with *sfGFP* gene). The sequence coding sfGFP was exchanged for gene coding mVenus using Gibson	M::mVenus<br>Amp^R<br>This work

	assembly with primers PF1, PR2 to amplify the backbone on p24MG2 template, and PF3 and PR4 with pKD3mVenus as source of gene coding fluorescent protein.	
p24R-mVenus	Based on p24RG2 (pBAD24 derivative carrying the sequence coding R.Csp231I in fusion with *sfGFP* gene). The sequence coding sfGFP was exchanged for gene coding mVenus using Gibson assembly with primers PF1, PR2 to amplify the backbone on p24RG2 template, and PF3 and PR4 with pKD3mVenus as source of gene coding fluorescent protein.	R::mVenus<br>Amp^R<br>This work

Strains

MG1655	K-12 F– λ– *ilvG– rfb-50 rph-1*	
MG1655 F^+	K-12 F– λ– *ilvG– rfb-50 rph-1* F^+	
MG1655 Δrac	as MG1655, but deletion of entire rac locus (about 21kbp)	Guo et al., 2014

Primers list

truekate1	(BstBI) **GG linker**<br>GGTTCGAA**GGTGGT**ATGGTTAGTAAAGGAGAAGAAAATAAC
revKateR	(StuI)<br>ACTAGGCCTCCTTTCGTTTTATTTGATGCCTCTAG
pFMpKD3	(NsiI)<br>CCTGATGCATAGATGGATATGAAATCGTAGAGC
pRMFULLpKD3	(NsiI) **GG-linker**<br>CGCGATGCATGC**ACCACC**ATCAAATAAAGATATAGTTTG
PFMFG1	(PstI)<br>AGTCTGCAGAGATGGATATGAAATCGTAGAGC
PRMFG2	(HincII)<br>AATGTTAACCTACTTGTACAGCTCGTCCATGC
PRC2	(BamHI)<br>CGAGGATCCTGATAAGCTGTCAAACATGAG
PF18Rfull1	(BstBI)<br>TACGCAAGTTATAGTAACCGTTTCG
PR18Rfull2	(BstBI)<br>TTATTCGAATAAACGATAAGAGTGTCGAAG
PF18mutR1	GGAGAAAATATTATTGTTGCCGCTGCTAAGTCTTACCGTTTAG
PR18mutR2	CTAAACGGTAAGACTTAGCAGCGGCAACAATAATATTTTCTCC
C23	AAAGAATTCATTTTTATATCAG
qR-REV	ATTTGGAGCTGCCTGACTT
PF24MGFP3	GTCCCATGGATGCGAAAGCAGTTATTAATA
PR24MGFP4	ATCTGCAGTAAAACGAAAGGAGGAACCTA
PF1	CGGTATGGACGAACTGTATAAATAATAAGCATGCAAGCTTGGCTG
PR2	GTTCTTCACCTTTGCTCATAAATTCGCCGCTGCCCG
PF3	GCAGCGGCGAATTTATGAGCAAAGGTGAAGAACTG
PR4	ACAGCCAAGCTTGCATGCTTATTATTTATACAGTTCGTCCATAC

Supplementary Figure S1. Test of the methylation activity clearly confirms the full activity of the MTase::sfGFP fusion protein.

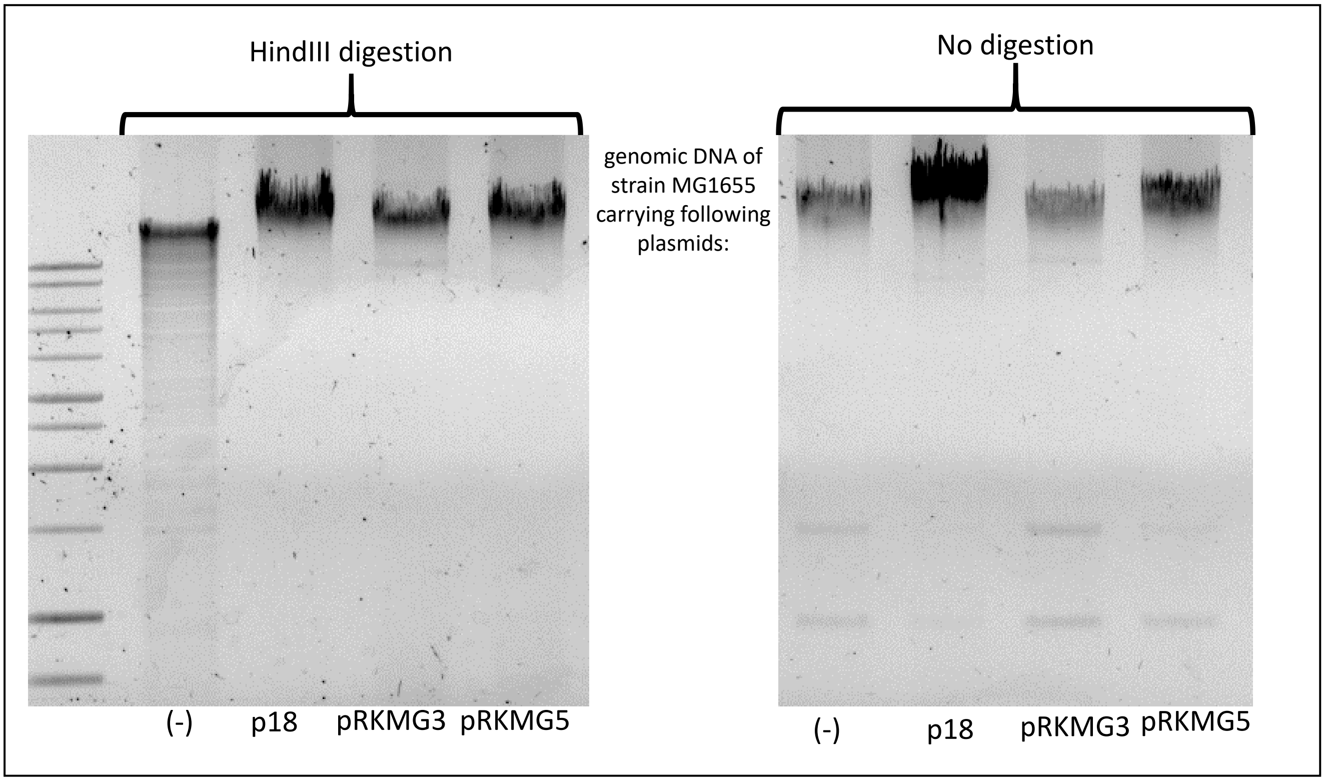

MTase protection activity assay

The MTase protection test analyzed the methylation status of selected HindIII sites on the *E. coli* MG1655 chromosome carrying the gene for fusion protein MTase::msfGFP, in comparison to WT MTase gene. Genomic DNA was isolated using a kit (A&A Biotechnologies). Then, its digestion with HindIII restriction enzyme was performed and the reaction products were loaded onto a 0.8% agarose gel and electrophoresed, with visualization following ethidium bromide staining and UV illumination. The level of genome degradation was evaluated using the controls. Genomic DNA isolated from cells carrying wild type Csp231I R-M system (p18) or not (MG1655 WT) was used as controls.

Supplementary Figure S2. Level of intensity for fluorescent fusions REase::mKate (red) and MTase::mGFP (green) in the *E. coli* cells infected with recombinant M13 bacteriophages carried operon of R-M Csp231I with C gene (RM3 C+) or without C gene (RM5 ΔC).

The fluorescence was measured 3 hours of post-infection time as detailed in Method section of the main manuscript.

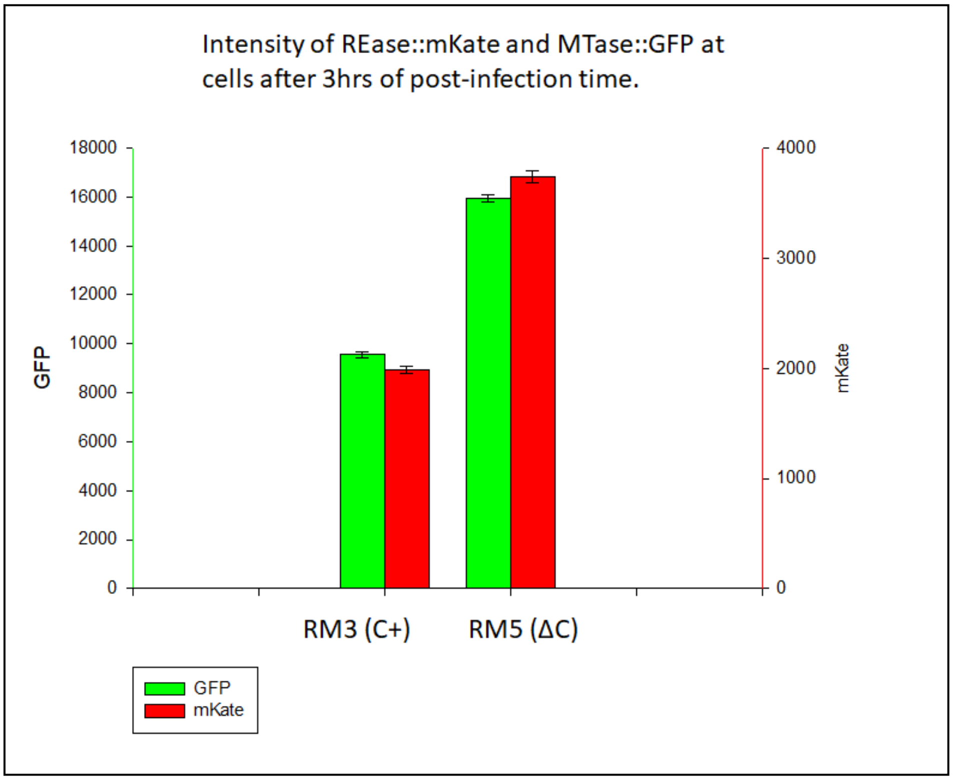

Supplementary Figure S3. The presence of mKate gene expression alone did not interfere with expression of REase::mKate fusion gene.

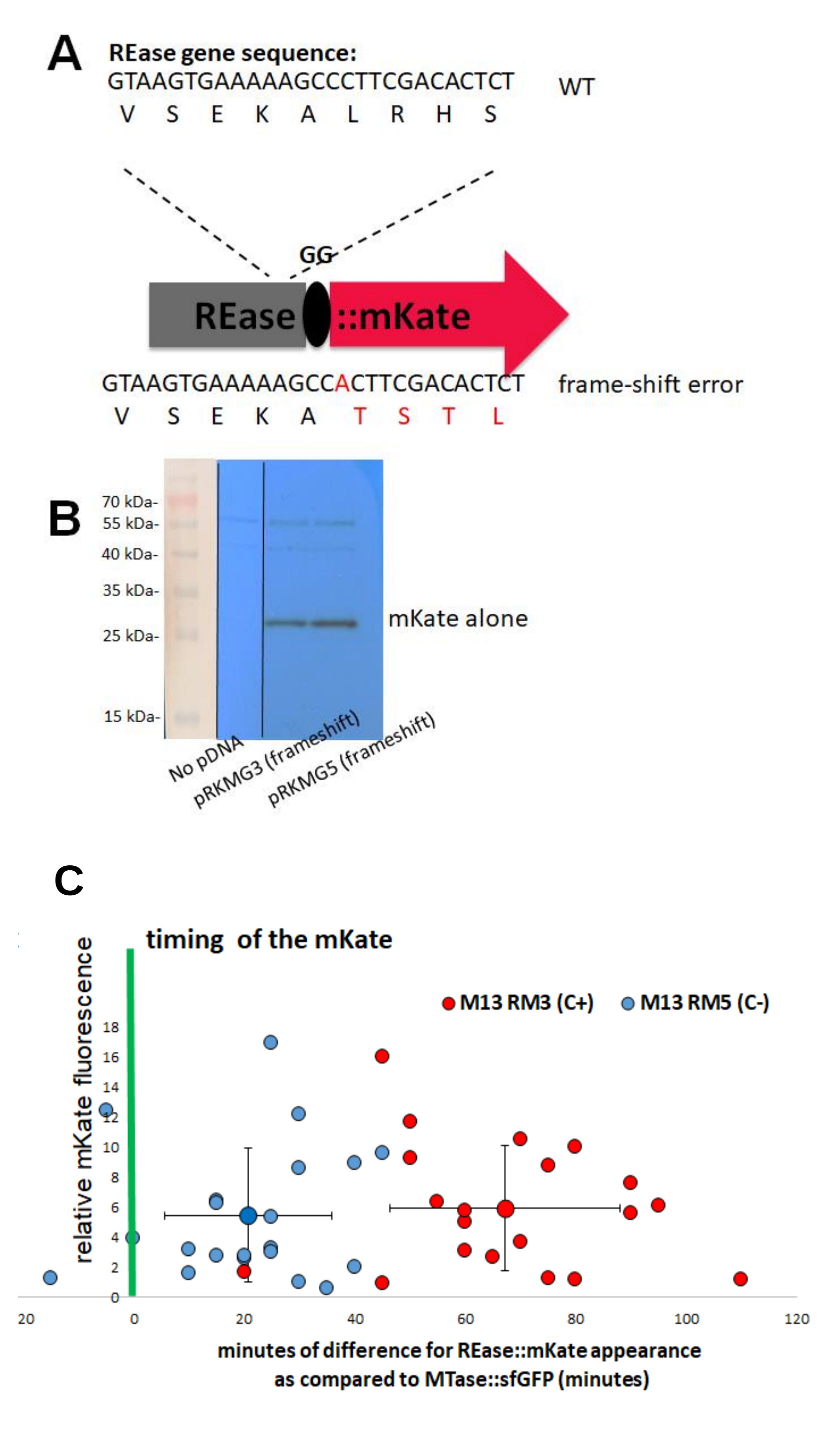

As a control, we tested whether the additional presence of mKate gene expression in both variants could interfere with the correct interpretation of the results of REase-expression (Figure 2C).

We used the recombinant M13 phages, as at M13RM3 (C+) and M13RM5 (ΔC), where the frameshift mutation was introduced into REase gene (Figure S3A) to remove the fluorescent signal from fusion protein and to test only mKate expression (as shown at western blots at Figure S3B). Infection with such REase-frameshifted recombinant M13 showed first detection of red fluorescence beyond the time range measured for M13RM3 and M13RM5, with REase timing about 67 min after MTase for the C+ variant, and 20 min after MTase for the ΔC variant (Figure S3C). So, there was still a REase time shift, which relied on a genetic operon design, and not simply on the REase gene being situated on the phage vector.

Supplementary Figure S4. Production of fusion proteins Csp231I restriction endonuclease or DNA methyltransferase with mVenus was confirmed using antibodies against green fluorescent protein (GFP) on cell extracts from *E. coli* carrying p24R-mVenus or p24M-mVenus, respectively. The fusion proteins were used in single-cell tracking analysis shown in Figure 6. As a positive control cell extract with plasmid carrying mVenus gene was used, while cells without plasmid served as a negative control.

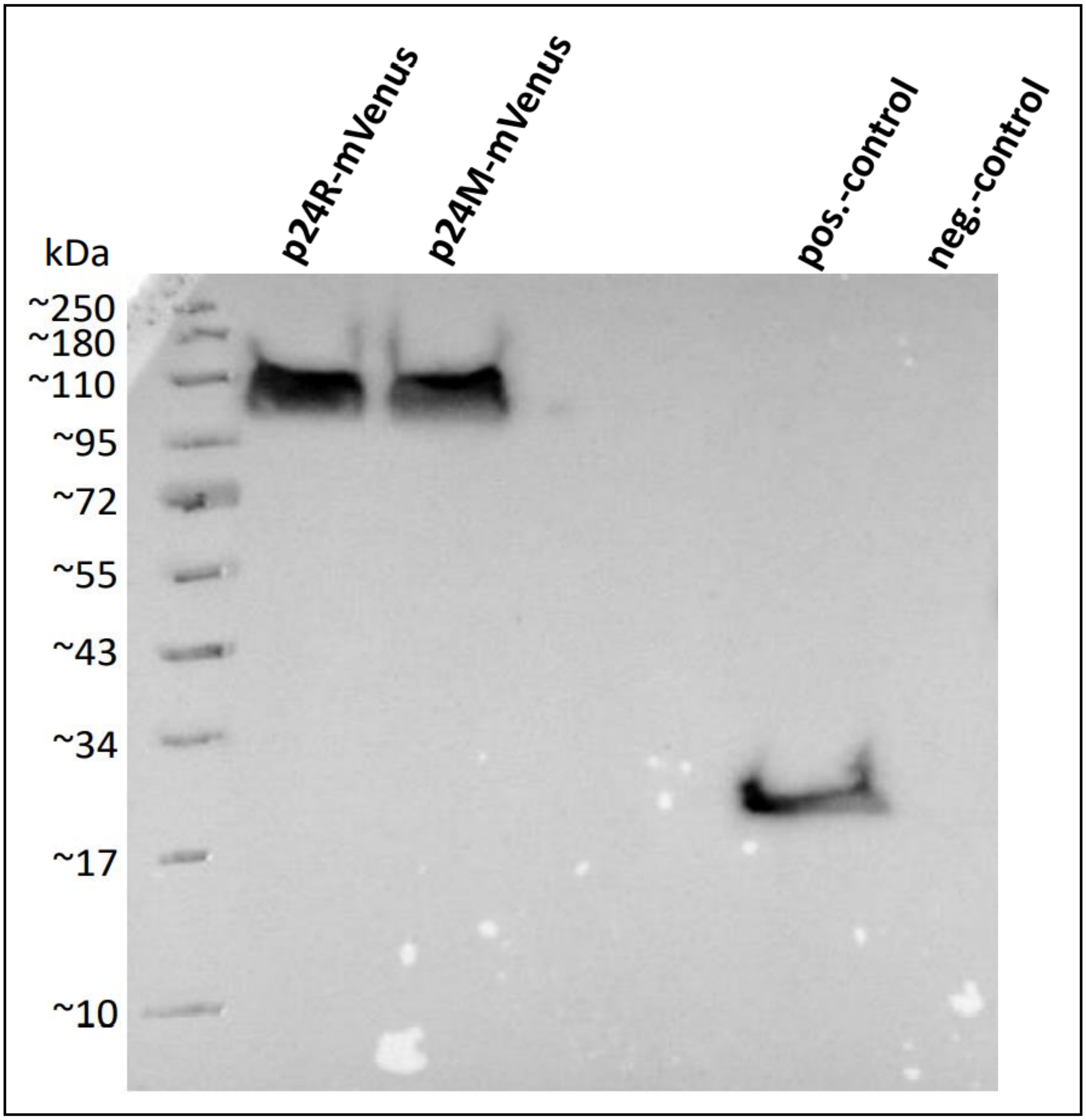

Supplement: gkab183_Supplemental_File [file gkab183_supplemental_file.pdf]
